# Functional Analyses of *NSF1* in Wine Yeast Using Interconnected Correlation Clustering and Molecular Analyses

**DOI:** 10.1371/journal.pone.0077192

**Published:** 2013-10-09

**Authors:** Kyrylo Bessonov, Christopher J. Walkey, Barry J. Shelp, Hennie J. J. van Vuuren, David Chiu, George van der Merwe

**Affiliations:** 1 Department of Molecular and Cellular Biology, University of Guelph, Guelph, Ontario, Canada; 2 School of Computer Science, University of Guelph, Guelph, Ontario, Canada; 3 Wine Research Centre, University of British Columbia, Vancouver, British Columbia, Canada; 4 Department of Plant Agriculture, University of Guelph, Guelph, Ontario, Canada; UGent / VIB, Belgium

## Abstract

Analyzing time-course expression data captured in microarray datasets is a complex undertaking as the vast and complex data space is represented by a relatively low number of samples as compared to thousands of available genes. Here, we developed the Interdependent Correlation Clustering (ICC) method to analyze relationships that exist among genes conditioned on the expression of a specific target gene in microarray data. Based on Correlation Clustering, the ICC method analyzes a large set of correlation values related to gene expression profiles extracted from given microarray datasets. ICC can be applied to any microarray dataset and any target gene. We applied this method to microarray data generated from wine fermentations and selected *NSF1*, which encodes a C_2_H_2_ zinc finger-type transcription factor, as the target gene. The validity of the method was verified by accurate identifications of the previously known functional roles of *NSF1*. In addition, we identified and verified potential new functions for this gene; specifically, *NSF1* is a negative regulator for the expression of sulfur metabolism genes, the nuclear localization of Nsf1 protein (Nsf1p) is controlled in a sulfur-dependent manner, and the transcription of *NSF1* is regulated by Met4p, an important transcriptional activator of sulfur metabolism genes. The inter-disciplinary approach adopted here highlighted the accuracy and relevancy of the ICC method in mining for novel gene functions using complex microarray datasets with a limited number of samples.

## Introduction

Microarray technology is commonly used to simultaneously monitor genome-wide gene transcription levels in a given organism. Large amounts of data generated by a few microarrays with thousands of features (i.e. genes) are complex to analyze. In time-course experiments each gene’s expression profile is treated as a vector of expression values (i.e. time series). Co-expressed genes are characterized by common expression patterns and often either share common biological function, participate in common biological pathways and/or respond to the same environmental variables [1-3]. This information can be used to predict and validate novel functional roles for unknown or poorly characterized genes. The analysis of microarray datasets has been a focus of data mining, statistical and systems biology research strategies, leading to the development of an array of data analysis approaches, including correlation clustering [4].

**Figure 1:**
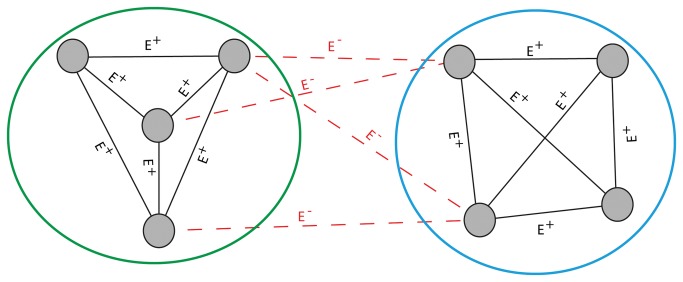
Illustration of the Correlation Clustering using an example of the graph *G* with {+} and {-} edges colored in black and red respectively. In graph *G* the gray circles refer to nodes (e.g. gene names) and connecting lines to edges (E) with {+} and {-} values. Green and blue circles represent putative clusters.

Our methodological extension to correlation clustering applies graph theory to analyze data that could be converted to an undirected graph (*G*), which typically consists of nodes (*N*) connected by edges (*E*). Given *G* with positive (*E* ^+^) and negative (*E* ^-^) edges representing similarities and dissimilarities among nodes, respectively (Figure 1), correlation clustering seeks to partition nodes into clusters. The number of *E* ^+^ (i.e. similarities) is maximized, and the number of *E* ^-^ (i.e. dissimilarities) is minimized within each cluster [4]. This type of clustering, with some modifications, has been used successfully to cluster genes based on similarity and dissimilarity of their respective expression profiles [4-6]. Compared to other partitioning clustering techniques, such as the popular *k*-means, correlation clustering does not require *a priori* specification of the number of clusters to partition the given data. This makes correlation clustering particularly attractive for the analysis of complex datasets where the data structure is not necessarily known, as is the case for genome-wide expression data.

The main aim of clustering is to compress and extract useful information from vast amounts of data. All clustering approaches therefore aim to partition data into arbitrary sub-groups (i.e. clusters) based on a defined similarity or distance measure. For example, genes sharing a similar biological function can be grouped together into discrete sub-groups. To assign a particular datum to a particular cluster, that datum must be most similar to existing objects within the cluster and least similar to other objects assigned to other clusters.

**Figure 2:**
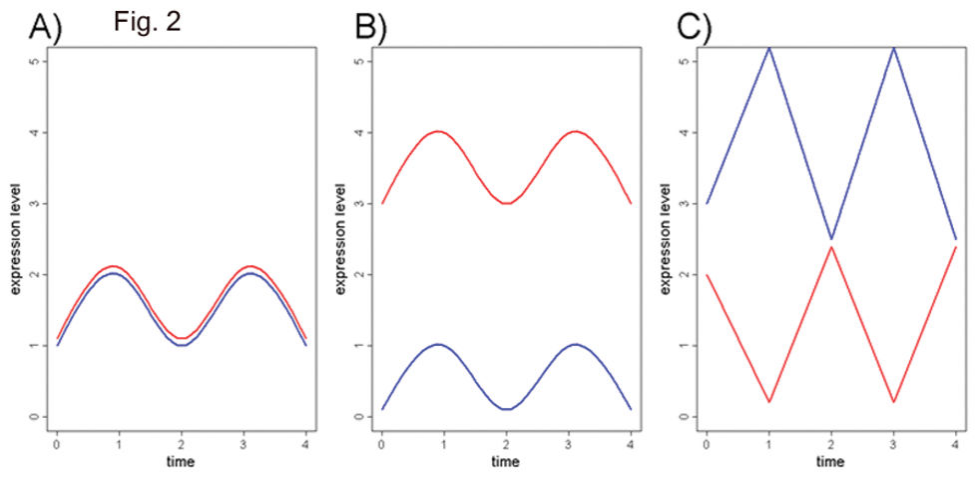
Illustration of expression profile of *x* and *y* with following patterns: A) *x* and *y* have both high similarity based on absolute difference and *r*(*x*,*y*); B) *x* and *y* have low similarity based on absolute difference but high *r*(*x*,*y*); C) *x* and *y* have very low similarity based on absolute difference but high negative *r*(*x*,*y*).

Similarity is determined by using a well-defined measure. For example, *x* = (*x*_*1*_ *,x*_*2*_ *… x*_*n*_) and *y* = (*y*_*1*_ *, y*_*2*_ *… y*_*n*_) are expression instances of two genes in a given cluster with the similarity *s*(*x,y*) existing between them (e.g. Euclidian distance). However, to cluster *x* and *y* while assuming common, possibly unknown, causation and interdependency, the more sensible measure of similarity would be correlation *r*(*x*,*y*), which assesses a common trend (increase or decrease) between *x* and *y* instances rather than differences in absolute values. If two expression instances *x* and *y* have similar shape and similar absolute values, both *s*(*x*,*y*) and *r*(*x*,*y*) display a high degree of similarity (Figure 2A). Nevertheless, common similarity measures *s*(*x*,*y*) fail to recognize two expression profiles (*x* and *y*) that have similar shapes, but different absolute expression values (Figure 2B). In addition, if profiles *x* and *y* are negatively correlated, they have inverse profiles with very low relative similarity value *s*(*x*,*y*), but highly negative *r*(*x*,*y*) value (e.g. *r* = -1) (Figure 2C).

Here, we applied our Interconnected Correlation Clustering (ICC) method to two time-course microarray datasets to find the largest interconnected gene cluster centered on a pre-selected *Saccharomyces cerevisiae* target gene. The microarray data were generated during the wine fermentation process and the pre-selected gene was *NSF1*, a poorly characterized gene previously identified to be activated during fermentation [7]. *NSF1* encodes a C_2_H_2_ zinc finger transcription factor (TF) that contains a typical ~30 amino acids DNA binding domain with two cysteines and two histidines tetrahedrally coordinated to a central Zn^2+^ ion [8]. The Nsf1p DNA binding sequence was identified as 5’-CCCCT-3’ [9]. This motif corresponds to stress response element sequences (STREs) found in promoter regions of genes involved in the general response of yeast to environmental stresses, such as the heat shock protein (HSP) genes, trehalose biosynthetic genes, and genes needed to combat oxidative stress [9]. To this end, *NSF1* was previously shown to be needed for the response of yeast to high osmolarity and poor quality carbon sources [8].

Fermentation is a complex process that subjects yeast cells to an array of environmental stresses including nutrient deprivation, low pH, hyperosmotic stress, and ethanol toxicity as the fermentation proceeds. The adaptation of wine yeast to fermentations is complex and is characterized by significant changes in gene expression [7,10]. Marks et al. identified 232 genes that were significantly induced (from 4 to 80 fold compared to their basal expression) during the fermentation process [7]. These genes were collectively termed the Fermentation Stress Response (FSR) genes and included *NSF1* [7]. This result suggested *NSF1* is involved in the FSR. In addition to the known participation of *NSF1* in carbon and energy metabolism, nutrient adaptation, and response to hyperosmotic stress [8], the ICC method proposed here suggested the involvement of *NSF1* in sulfur metabolism, vesicle trafficking, cell cycle control, and regulation of protein synthesis during fermentation. In particular, we provide evidence that confirms the role of *NSF1* as a negative regulator of sulfur metabolism genes, thereby validating the ability of the proposed ICC method to identify biologically relevant predictions.

## Methods

### Genome-wide expression datasets used

**Figure 3:**
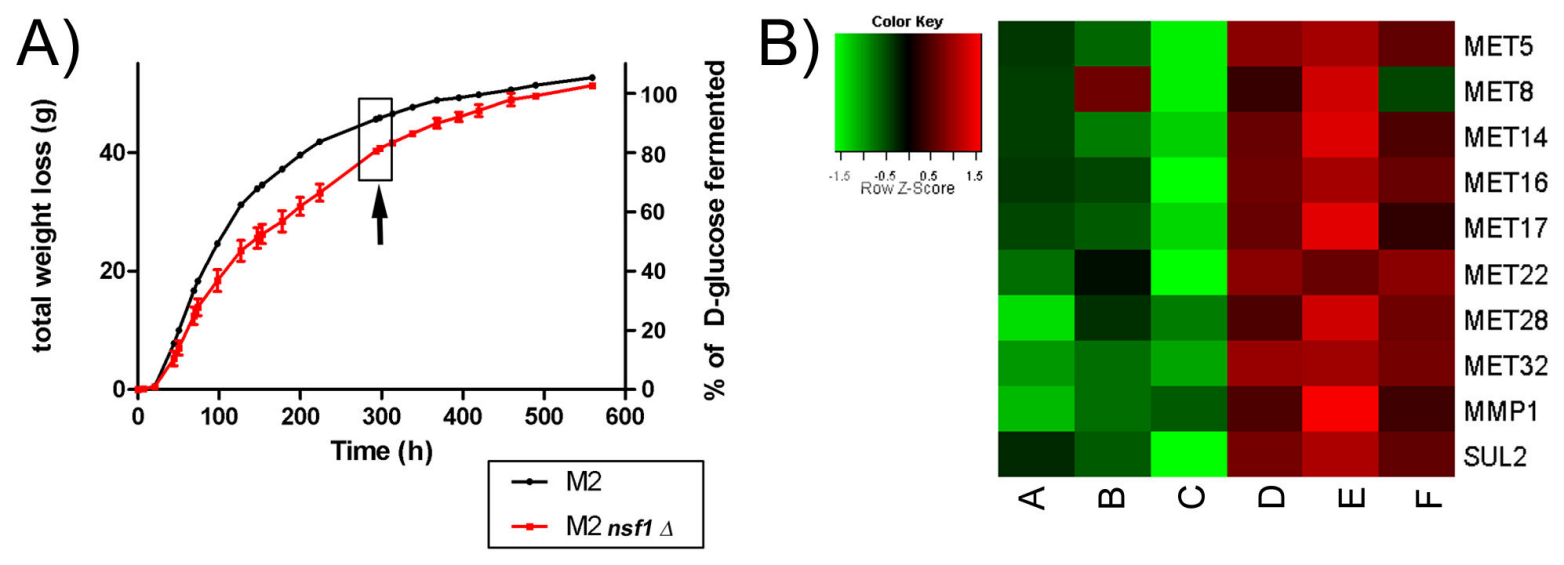
Fermentation profile and the impact of *NSF1* on the controlled expression of sulfur pathway genes during Riesling fermentation. **A**) Fermentation profile of the M2 and M2 *nsf1∆* in Riesling grape must measured by the amount of culture weight lost due CO_2_ production. The arrow shows the datum after 85% sugar fermentation, the time at which DEGs were determined. Error bars represent standard deviation (SD). **B**) The heatmap of the sulfur related genes from the MFD dataset microarray expression data corresponding to fermentation of 85% of the sugars. The expression values were normalized for each gene by converting them into *z*-scores (absolute expression value – mean expression across all samples / SD across all samples) in order to ensure median expression value of zero for each gene across all samples. M2 triplicate samples are represented in columns A-C and those for M2 *nsf1∆* in columns D-F.

The ICC method was applied to two microarray datasets; both sets were generated by the fermentation of Riesling grape juice with two different wine yeast strains. The first dataset, designated the M2 Fermentation Dataset (MFD), was obtained in this study by using the industrial M2 *S. cerevisiae* strain to ferment 2 L Riesling grape must, in biological duplicate, in flasks capped with air locks without shaking for 15 days at 18 °C. The progress of the fermentations were monitored by measuring the concentrations of D-glucose using the Megazyme® D-Glucose HK kit (Xygen Diagnostics) according to the manufacturer’s specifications and by measuring the amount of weight loss during fermentation due to CO_2_ production. Samples were collected at three time points; 24 h post-inoculation, and when 20% and 85% sugars were fermented. Figure 3A shows the fermentation profile. Sampling points correspond to three stages of fermentation: the initial stage (24h ~5% sugars fermented), exponential or active stage (20% sugars fermented), and the final stage (85% sugars fermented). Thus, monitoring the percentage sugars consumed by the yeast allows for the monitoring of fermentation progression. The yeast cells were harvested, washed and total RNA was isolated [7] and cleaned using Qiagen™ RNeasy columns for microarray analysis. Thus, the MFD dataset was generated specifically for this study and was not published before. Gene expression data were obtained with Affymetrix Yeast 2.0 arrays using previously described methods [7]. The raw data were first normalized using the Robust Multi-array Analysis (RMA) algorithm. In addition, the *S. pombe* and other non-informative service probes were masked. The filtered data contained expression data corresponding to 5667 genes. 

The second microarray dataset was generated and published by Marks et al. (2008) as an investigation into the adaptation of the Vin13 wine yeast strain to fermentation conditions [7]. The VFD dataset was obtained via Gene Expression Omnibus database (Reference #: GSE8536). It represents the adaptation of the global transcriptome profile of Vin13 *S. cerevisiae* wine yeast strain generated during a 15 day fermentation in Riesling grape must. For the sake of simplicity we designated this dataset the Vin13 Fermentation Dataset (VFD). This expression data consisted of a total of 21 microarrays; 3 microarrays at 7 time points. The time points corresponded to 1, 12, 48, 60, 120 and 340 h (corresponding to 0%, 0.5%, 18%, 32%, 64%, 100% total sugars fermented) after inoculation of the Riesling grape must. Global gene expression was measured using Affymetrix™ Yeast Genome S98 chips with 9335 probes, but only 6300 probes were mapped to the verified Open Reading Frames in the Saccharomyces Genome Database (SGD).

To identify differentially expressed genes (DEGs) dependent on *NSF1* in the MFD dataset, the M2 *nsf1::KanMX*/*nsf1::KanMX* homozygous mutant (M2 *nsf1∆*) was used for parallel fermentations along with the M2 strain as outlined above. Samples were collected at 85% sugars fermented (Figure 3A). The DEGs at this time point were identified using two-sample, two tailed unpaired t-test at 95% confidence level assuming unequal variances between M2 and M2 *nsf1∆* sample groups. 

The M2 Fermentation Dataset (MFD) expression data are available at ArrayExpress (Accession #: E-GEOD-34117) or GeneOmnibus (Accession #: GSE34117) repositories. The Vin13 Fermentation Dataset (VFD) [7] raw expression data can be accessed through GeneOmnibus (Accession #: GSE8536).

### The ICC Method and the formation of the largest interconnected correlated gene cluster

**Figure 4:**
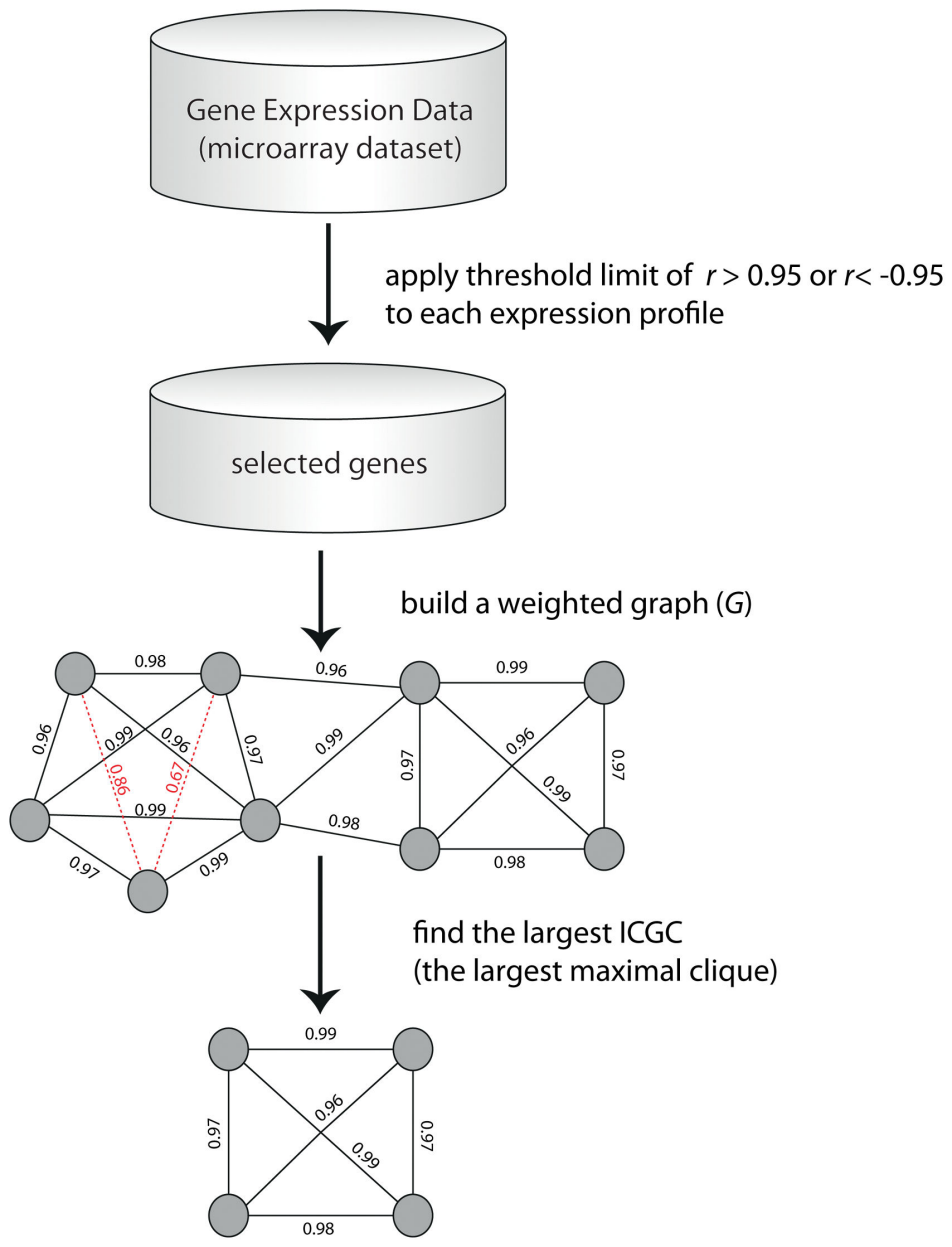
The overall ICC method workflow culminating with formation of the largest Interconnected Correlation Gene Cluster (ICGC).

The proposed ICC method uses both correlation clustering to represent data as a weighted undirected graph (*G*) and the Born-Kerbosch heuristic algorithm [11] to search for the largest maximally interconnected correlated gene cluster (ICGC) representing a tight cluster of co-expressed genes conditioned on the target gene *NSF1* (Figure 4). Importantly, this method emphasizes properties of edges (i.e. similarity between expression profiles) summarized by the Pearson’s correlation coefficient (PCC or *r*) rather than the functional properties of nodes (e.g. gene family, gene function, etc). 

Our ICC method is outlined by the following steps:

**Step 1:** Compare each individual gene expression profile to the target gene (e.g. *NSF1*) represented by the PCC_target gene_ statistic.

**Step 2:** Select genes highly positively and negatively correlated to the target gene passing the threshold *r*<-0.95 or *r* >0.95. Store the selected genes in the *select*_*array*.

**Step 3:** Build a weighted graph (*G*). Assign *E* ^+^=1 if the PCC value between corresponding vertices meets the threshold of *r*<-0.95 or *r* >0.95; otherwise assign *E* ^-^ = 0.

**Step 4:** Find the maximally interconnected sub-group of nodes, the ICGC, in *G* using the Born-Kerbosch heuristic algorithm.

The pseudo-code in the Supplementary Information (Figure S1) describes the ICC method in greater detail where *X*[*gene*_*i*_] and X[*target*] represent a gene expression profile of *ith* and *target* genes across *t* time points.

The resulting ICGC represents co-expressed genes that are conditioned on *NSF1*, all sharing very similar expression profiles defined as *E* ^+^ edges with positive and negative PCC values that fall within the *r* < -0.95 or *r* > 0.95 threshold. Thus, the resulting ICGC has none of the *E* ^-^ edges that have PCC values outside the aforementioned threshold. Since Pearson’s *r* values (PCC) are not normally distributed, it was necessary to convert these values to a statistic with approximately normal distribution, such as *z* scores, to select a statistically significant threshold. All *r* values calculated from step 1 of the ICC method were converted to their corresponding *z*-scores according to the standard *r* to *z* Fisher’s transformation using the following formula:

$$z=0.5*\log\frac{1+r}{1-r}$$

**Figure 5:**
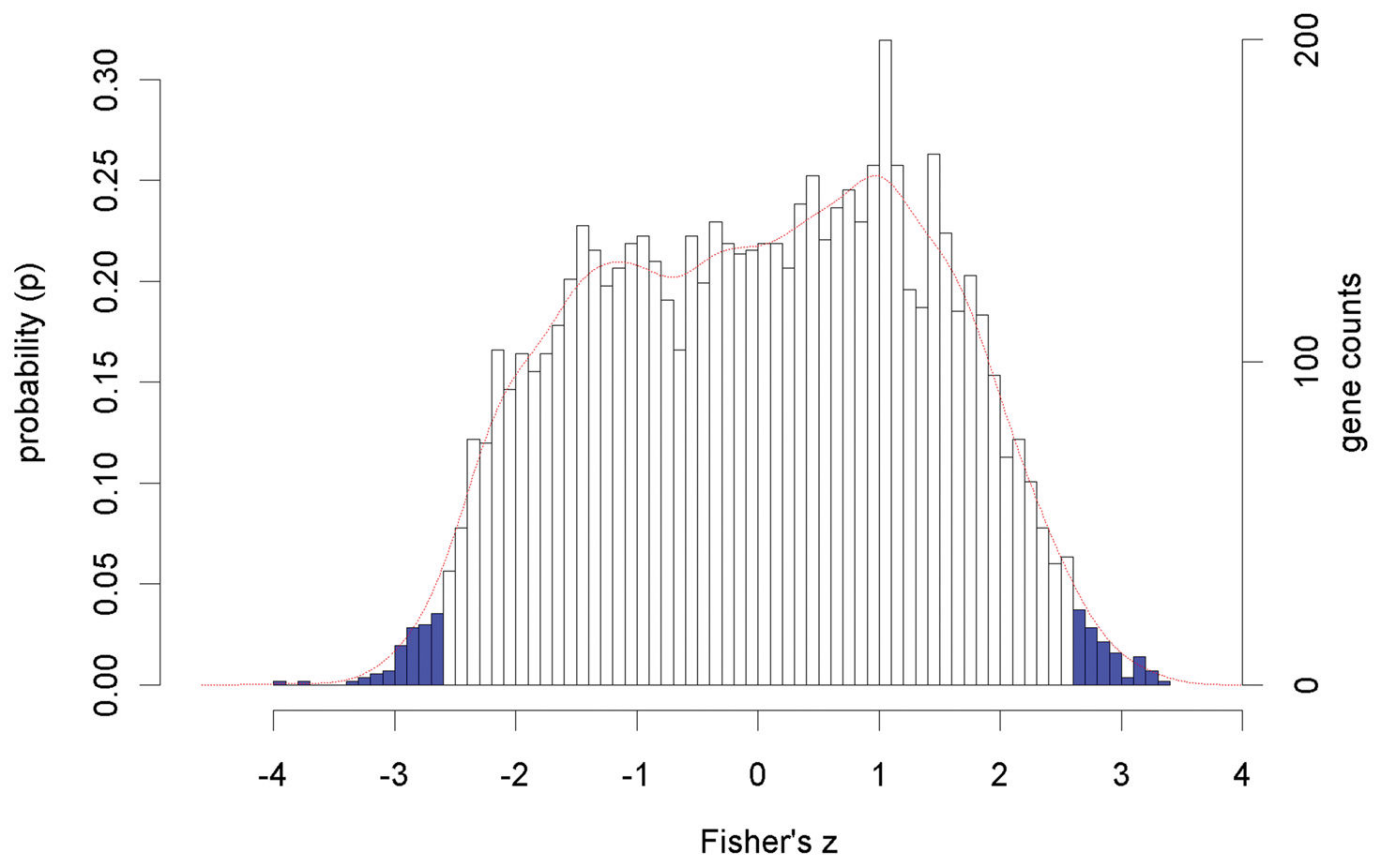
Distribution of the *z*-scores corresponding to all genes (a total of 5667 genes) with the empirical probability density function plotted as a red line. The probability (*p*) corresponds to probability density function of finding a particular *z*-score at a particular value. The calculated *z*-scores were derived from *r* values. These values were obtained from a comparison of the *NSF1* expression profile to that of every other gene in the dataset (r). The blue bars correspond to critical regions at 0.95<p<0.05 (α=0.10) based on the empirical distribution of z-values (critical z-score value 0073: -2.15, 2.15). The skewness of the z-scores was found to be -0.07084± 0.0637 confirming approximately symmetric distribution; the standard error of skewness (SES) was found to be 0.03253.

The population of *z*-scores with a variance (σ_z_) of 1.89 was plotted (Figure 5). The distribution of the *z*-scores was assumed to be approximately normal as seen from the shape of the histogram and probability distribution function (PDF). To confirm, we calculated the skew to be only -0.07084± 0.0637 indicating a slight shift to the left. Given that skew values falling within the range of -0.5 to 0.5 are considered to be reflective of approximately normal distributions, the previous assumptions are correct [12]. To further analyze the distribution, we calculated kurtosis obtaining a negative value of -0.93 which indicates that *z*-distribution has flatness and “light tails” with relatively lower than normal number of observations at its extremes. This means that the number of extreme values was rather limited. In addition, the calculated Shapiro-Wilk (SW) normality test statistic W = 0.9806 at p-value = 0.087 and Kolmogorov-Smirnov (KS) two-sided test statistic at D = 0.120 at p-value = 0.048 indicated that distribution could be considered as approximately normal although at the limit of normality at α=0.05. The obtained 0.95<*p*<0.05 according to the empirical PDF corresponded to *z*-scores of -2.15 and +2.15, translating to *r* of -0.97 and +0.97. According to our empirical *z*-distribution, the *r*=0.98 corresponds to p=0.034 while *r*=0.95 to p=0.093. Due to the relatively small size of the dataset (12 samples), the limited number of time points, and the possibility of having false negatives at *r*<-0.98 or *r*>0.98, and *z*-scores distribution with relatively “light tails” based on kurtosis value, the correlation threshold was lowered to *r*<-0.95 or *r*>0.95 which is slightly outside of the classical statistical two-tailed α=0.10 threshold. While being aware of the risk of getting a higher number of false positive hits at lower threshold, our goal is to get some true positives in presence of false positives. In addition, the obtained ICGCs for *NSF1* at -0.95 >*r*>0.95 and -0.98>*r*>0.98 had 77.3% overlap in gene composition. This shows a low threshold impact on the final results with the ICGC preserving the initial core. Thus, selection of the threshold is mainly based on the desired size of the ICGC and biological context. We recommend calculating the percent overlap between ICGCs under different thresholds to judge its impact on reliability and robustness of the final results. We recommend selection of the threshold between -0.95 >*r*>0.95 and -0.98>*r*>0.98.

To test the relative performance and reliability of the proposed ICC method, two microarray datasets were used to find novel *NSF1* gene roles under fermentation conditions. *NSF1* was previously found to be a regulator of gene expression when the yeast uses poor carbon sources and to participate in adaptation responses to hyperosmotic and nutrient stresses [8]. These functions were used as qualitative performance measures of the ICC method. The complete list of ICGCs genes generated for two datasets, MFD and VFD, are listed in Tables S1 and S2, respectively.

### Functional Enrichment Analysis by GO terms

Genes identified using ICC were analyzed for functional enrichment using the Gene Ontology (GO) database and hypergeometric probability distribution function (PDF) to additionally confirm previous functional results. The GO database is a hierarchical acyclic graph composed of GO terms as vertices where the top levels contain general GO terms and the very bottom levels contain the more specific terms [13,14]. Thus, each gene could contain more than one associated GO term. In this study, we selected the GO database related to ‘biological processes’.

To test enrichment of the GO terms while accounting for the GO graph structure we used the *elim* algorithm based on Fisher’s exact test. The algorithm is implemented as a topGO library for R [15]. Briefly, each GO term is being annotated using two gene sets (genes part of ICGC and entire genome). Thus, each gene is being categorized based on two criteria: a) occurrence in the ICGC and genome-wide lists; b) ability to map or not to a given GO term. The GO tree is traversed from the lowest level (leafs) to the highest (root) calculating Fisher’s exact test only on the remaining genes that were not eliminated from the previous steps. Thus, the *elim* method is superior to traditional Fisher’s exact tests performed on 2 by 2 contingency tables in that it performs multiple-testing corrections while accounting for the GO structure. The node (GO term) is kept if its resulting p-value < 0.01 after Bonferroni correction which was performed by multiplying the obtained raw p-value by the total number of nodes in the graph mapping to at least one annotated gene [15]. The parameters for the *elim* algorithm were as follows: 1) minimum 20 genes should map to a given GO term for it to be kept in the GO graph; 2) Fisher’s exact test is calculated for each GO term; 3) entire GO graph is considered from the lowest to the highest level (bottom up); 4) cut off p-value is fixed at 0.01 for each GO term considering the Bonferroni correction as per [15]. Those GO terms with an adjusted p-value < 0.05 were considered to be statistically significant and occur non-randomly in the ICGC. The complete tables of top 300 GO terms output by functions of the topGO library [15] are shown in Tables S3 and S4 corresponding to the MFD and VFD datasets, respectively.

While it is important and statistically sound to correct p-values for multiple testing effects to reduce false positive hits (Type I error), current multiple-testing correction techniques are not optimal. Specifically, classical multiple-testing correction techniques (e.g. Bonferroni correction) are very restrictive, they do not adequately account for the GO graph structure and some GO terms are not associated with any genes. Thus, classical multiple-testing correction techniques often produce overly conservative adjusted p-values that can lead to the loss of biologically relevant information. Thus, it can still be informative to consider GO terms with adjusted p-values > 0.05. 

To heuristically assess the extent of the functional enrichment of a given functional category of the ICGC genes shown in Tables 1 and 2, we performed separate GO functional enrichment analyses for each category using the *elim* algorithm with exactly the same settings as used for the GO enrichment analysis of the largest MFD and VFD ICGC. The average p-value for each category was determined by calculating the mean of the GO terms p-values. Only GO terms mapped to category genes with minimal p-value (each gene can be annotated to several GO terms) were selected for calculation of the final average category p-value. Thus each category gene was represented by one GO term with lowest possible p-value.

**Table 1:** Selected genes from the largest ICGC by category from the MFD dataset.

**Functional Category**	**Representative Genes**	**Average p-value**
stress response	*ATP1, VMA1, CIT2, **HAL1, PCK1**, STM1, **SLX4**, HMF1, **APJ1**, AIM14, YVC1, GSH2, **GSH1**, **MIG3**, **SSC1, FRT1**, HKR1,IZH13*	0.0235
cell cycle control	*Figure **1****, KEL2, MPS1, RAD24, VHS1, BAR1, SPO22, SSP2, SSP1, SPO11, HOP2**, CDC28*	0.013
carbohydrate metabolism/energy metabolism	***QCR8**, VMA5**, MTH1, KGD2, ISA1**, CPS1, **PDE1, MLS1, ATP18, ATP19, VMA11, YIA6, RIB1***	0.014
ribosome assembly/ protein synthesis	*RPS3, RPS13, RPL7A, RPS6B, RPS2, RPS16B, RPS21B, RPS9B, RPS23B, RPS0B, RPS22B, RPS8A, RPS7A, RPS24B; RPL43B, RPL2A, RRP5, RPL18A, PRE5, RPS17B, RPS5, RPL8A, RPS30A, RPL16A, RPS18B, RPL26A*	0.040
transcription / translation regulation	*ACS1, DED81, PMT4, **SPT2**, HTS1, SES1, BUR6, URA4, PRO3, THS1, ARC1, TEF4, CDC73, ADK1, TRP2, ARO8, IMD4, EGD1, TIF4631, GLN4, ILV2,CGI121, **STP1, SLU7**, MMF1, ARO4, ARG4, **PRP45**, URA5**, DDS1**, LYS2, POL5*	0.022
protein degradation	*PRE9, OLA1, **VID24**, **DAS1**, **SAN1**, UBA1, **YLR224W**, **PEX28**, **PIB1***	0.030
vesicle trafficking	*VPH1, CHC1, SEC 23, VAC8, SAM50, FEN1, EMP70, VPS75, TRX1,**BET1**, VPS1, **ATG23**, COG1*	0.0038
cell wall related proteins	*ROT2, KRE5, **SKG1**, PMT6, **GAS4***	0.0074
cell nucleus trafficking	*NUP192, NUP188, **NUP42**, NUP133, NUP82, KAP104*	0.0020
sulfur metabolism	*HOM2, **MET4***	0.0012

Note: ‘**Bolded**’ and ‘non-bolded’ genes are up-regulated (PCC > 0) and down-regulated (PCC < 0), respectively, at the end of fermentation (85% sugars fermented, which represents fermentation progression) with respect to the 24 h time point. A complete list of MFD ICGC genes is provided in Table S1. The average p-value corresponds to the average p-value of GO terms linked to the category genes (see Methods).

**Table 2:** Representative genes found in the largest ICGC from the VFD dataset.

**Functional Category**	**Gene Symbol**	**Average p-value**
Vesicle trafficking	*ERV41*, *SRP21*, ***VPS8***, *ARL1*, *NTF2*, *ARF1*, SEC *3*, ***UFD1***	0.0053
Post-translational protein modification	*PMT4*, *NAT5*, *SRP68*	0.011
Stress response	***RTC1***, ***RIM15***, ***RPN4***, ***MEP1***, ***GIP2***	**0.02**
Sulfur Metabolism	*SES1*, ***MET4***, *FSH3*, ***ARC1***, FOL *1*	0.0152
Ribosome Assembly / Transcription / Translation	*RPL34B*, *RPL22B*, *RPS5*, *RPS25A*, *PL17A*, *RPS0A*, *RPL13B*, *RPS13, RPL27A, RPS24A, RPS23A* ,*RPS7A, RPL3, IWR1, RPS1A, EGD2, DCD1, GET1, RPP1A, RPL31A*	0.036

Note: ‘**bolded**’ and ‘non-bolded’ genes are up-regulated and down-regulated at the end of fermentation (85% sugars fermented, which represents fermentation progression) with respect to the 24 h time point. A complete list of VFD ICGC genes is provided in Table S2. The average p-value corresponds to the average p-value of GO terms linked to the category genes (see Methods).

Subsequent sections analyze collectively each predicted functional category under common biological context of the datasets (i.e. fermentation conditions) in order to qualitatively validate the ICC method. 

### Validation of *NSF1* involvement in sulfur metabolism

#### Yeast strains and media composition

All the yeast strains used in this work are isogenic to the wine yeast strain M2 (Lallemand) and listed in Table S5. Mutant strains were generated by integrative transformation as previously described [36]. Primers to generate the integration cassettes are listed in Table S6. Each contained 70 nucleotides homologous to the native genomic DNA sequence flanking the targeted site of integration to facilitate homologous recombination. pFA6-natNT2 was used as template for generating the *MET4* disruption cassette [16]. Correct integration events were confirmed by PCR. All cell growths were performed at 30 °C with constant agitation unless otherwise stated.

#### RNA extraction for qRT-PCR analysis

The cells were grown in sulfur limited (S-) or sulfur rich (S+) medium detailed in Boer et al. [17] (Table S7). Briefly, over-night cultures of M2, M2 *nsf1*Δ, M2 *met4*Δ, M2 *met4*Δ*nsf1*Δ and strains were grown in Yeast Nitrogen Base (YNB) complemented with 2 mM methionine to account for the methionine auxotrophy of *met4*Δ strains. The cells were harvested, washed with dH2O and used to inoculate S- media; cultures were grown at 30 °C for 24 h. Total RNA was subsequently isolated using the previously described standard phenol-based RNA extraction method [18]. Total RNA was treated with DNAse I (Qiagen™DNAseI kit) and purified with Qiagen™ RNeasy spin columns as per the manufacturer’s instructions prior to qRT-PCR analysis.

#### Transcriptional analysis by qRT-PCR

The primers used for qRT-PCR are listed in Table S8. Primers with efficiencies of at least 75% were used. The obtained ΔCt values for five biological replicates were analyzed using the Pfaffl method [19]. The statistically significant changes in gene expression across two conditions were identified using the one-sample t-test with population mean of 1.0. The ratio of 1.0 between expression values originating from two different conditions highlights no change in gene expression. Thus, the expression ratio greater or lower than 1.0 between two strains refer to up-regulation or down-regulation of a given gene, respectively. Genes with expression values across two conditions with p<0.05 were considered statistically significant.

#### Nsf1-GFP localization

The subcellular localization of Nsf1-GFP was analyzed using the M2 *NSF1/NSF1-GFP-KanMX* strain transformed with pNIC96-mCherry-hphMX. Nsf1-GFP and Nic96-mCherry were detected with fluorescence microscopy. Nic96 is a nucleopore complex protein and therefore demarcates the nucleus. The strain was grown in sulfur rich (YNB S+) medium complemented with MgSO_4_, or sulfur limiting (YNB S-) medium devoid of MgSO_4_ (Table S7). Cells were grown overnight in YNB S+ or YNB S- medium and shifted to corresponding fresh YNB S+ or YNB S- medium. When these cultures reached exponential growth, they were divided in two, the cells harvested, washed with dH_2_O and transferred to fresh YNB S+ or YNB S- medium and incubated at 30 °C. Samples were collected at 0, 0.5, 1, 3 and 6 h post inoculation. Slides were prepared directly from the indicated cell cultures followed by immediate analysis using the 100× objective lens of a Nikon Eclipse E600 microscope. Images were recorded using a Coolsnapfx monochrome CCD digital camera (Roper Scientific) and processed using Metamorph (Universal Imaging, Version 5.0).

## Results

### MFD ICGC overview

The maximal ICGC conditioned on *NSF1* obtained after gene expression analysis of the six microarrays contained a total of 254 genes that were characterized through manual curation into 10 biologically relevant categories. The most representative genes of each category are shown in Table 1. The bolded genes show up-regulation or down-regulation towards the end of fermentation (corresponding to 85% sugars fermented, the last time point) as compared to the first time point during the initial stage of fermentation. The most prevalent functional categories represented by genes in the ICGC corresponded to biological processes related to: (1) transcription, translation and protein modification; (2) various stress responses; (3) cell cycle control; (4) ribosome assembly; and, (5) carbohydrate, energy metabolism and nutrient adaptation (Table 1). As expected, not all 254 genes in the ICGC had known biological functions. In addition, in comparison to the overall GO functional enrichment results of the MFD ICGC, similar enriched functions related to protein synthesis, transport and degradation, and nitrogen, energy and sulfur metabolism functions were observed (Table S3).

### VFD ICGC overview

The VFD ICGC for the dataset contained 83 genes that were functionally categorized (Table 2). Similarly to the MFD dataset, the main functional categories related to: (1) protein synthesis; (2) vesicle trafficking; (3) sulfur metabolism; (4) stress response; and (5), energy metabolism. 

The representative genes with known biological functions present in the VFD ICGC are shown in Table 2. Again, the protein synthesis and vesicle trafficking categories had the most genes with the majority of genes down-regulated towards the end of the fermentation (Table 2). Cell cycle and energy metabolism categories contained fewer genes than in the MFD dataset. The sulfur metabolism category had more genes than in the MFD dataset. The key regulator of the sulfur metabolism, *MET4*, was present in both datasets and was up-regulated towards the end of the fermentation (Tables 1 and 2). 

The most significant GO terms from the functional enrichment analysis of the VFD ICGC were related to protein and amino acid synthesis, nutrient utilization and energy metabolism, and stress responses to toxins (Table S4). Please note that p-values for corresponding GO terms are rather conservative due to multiple-testing corrections (see Methods). 

### Functional analysis of the MFD and VFD ICGCs in relation to *NSF1*

The results from the MFD and VFD datasets provided similar *NSF1* functional contexts, highlighting the robustness of the method. The genes in the two ICGCs represent functional neighbourhoods that allow predictions to be made as to the putative biological functions of *NSF1*. Interestingly, the genes within the respective ICGCs differed significantly (Tables 1 and 2), but there was little variation observed in terms of biological functions. This is not surprising as the datasets were generated with two different wine yeast strains, M2 and Vin13, fermenting two different vintages of Riesling grape must.

#### *NSF1* involvement in energy metabolism and response to nutrient limitation

*NSF1* could directly regulate energy metabolism genes that are part of the TCA cycle and ATP production pathways in response to nutrient limitation conditions as suggested by functional analysis of the genes present in the ICGCs. For example, *KGD2*, *YIA6* and *MLS1* were clearly functionally linked as genes needed for important steps in the TCA cycle (Table 1). Dihydrolipoyl transsuccinylase (*KGD2*) participates in the mitochondrial conversion of 2-oxoglutarate to succinyl-CoA, which requires NAD^+^ to be carried from the cytoplasm into the mitochondria by the transporter encoded by *YIA6* [20]. Malate synthase (*MLS1*) utilizes glyoxylate to produce malate, which in turn is converted into the TCA cycle intermediate, oxaloacetate. 

*ATP18*, *ATP19* and *ATP23* are part of the essential F_0_F_1_-ATP synthase complex that is located in the inner membrane of mitochondria; a proton gradient across the membrane is required to produce ATP molecules under aerobic conditions (Table 1). *ATP18* and *ATP19* represent the *j* and *k* subunits while *ATP23* is a metalloprotease required to process the *a* subunit [21]. All genes in the energy metabolism category were up-regulated towards the end of fermentation (i.e. 85% of sugars fermented), reflecting the increased energy demands of the yeast as nutrients were depleted and fermentation stresses were enhanced (Table 1). These observations were supported by the GO functional enrichment of the ICGC that highlighted nutrient-related processes such as the utilization of ATP (GO:0046034 p=0.0086)(Table S3) and energy production via H^+^ proton transport (GO:0015992 p=0.00346)(Table S3), nitrogen utilization and production of non-fermentable and fermentable sugars (GO:1901137 p=0.04525), and nutrient transport pathways (Table S3). Collectively, these data suggest the participation of *NSF1* in energy metabolism when nutrients become limiting.

#### *NSF1* could function in stress response toward the end of fermentation

*NSF1* is known to participate in the response of yeast to environmental stress, specifically salt stress [8]. In addition, *NSF1* was identified as one of the FSR genes in a Riesling fermentation [7]. To this end, the MFD ICGC identified genes known to participate in the stress response of yeast: *GSH1* and *GSH2*; *HAL1*; and *APJ1* (Table 1). γ-Glutamylcysteine synthetase and glutathione synthetase (*GSH1* and *GSH2*) are known components of the yeast stress response as they are involved in the production of glutathione, an essential thiol compound and reductant implicated in detoxification of toxic chemicals and combating oxidation damage by free oxygen radicals [22]. *APJ1* encodes a member of the Hsp40-family of chaperone proteins that interact with Hsp70 proteins involved in protein assembly and trafficking [23]. *HAL1*, which is involved in hyper-osmotic stress responses, decreases intracellular Na^+^ via interaction with Ena1p, a known target of the *NSF1* [8,24]. Furthermore this is supported by the GO term of the MFD ICGC related to regulation of cellular response to stress (GO:0080135 p=0.00143)(Table S3).

Further analysis of the VFD ICGC revealed strong correlation among *GIP2*, *RIM15* and *NSF1* co-expression (Table 2). The synthesis and accumulation of intracellular glycogen is one of the physiological mechanisms used by yeast to respond to environmental stress [25]. Glycogen metabolism is partially controlled by the actions of the protein phosphatase Glc7p and the PAS kinase Rim15p. Gip2p is a putative subunit of the protein phosphatase Glc7p involved in activating glycogen accumulation [26,27]. *GIP2* expression is induced by glucose limitation and ethanol shock [28,29]. In turn, *RIM15* encodes an effector kinase regulated by both the Target of Rapamycin (TOR) and RAS/cAMP/Protein Kinase A (PKA) signalling pathways to coordinate cell growth with environmental conditions. Environmental stress inactivates TOR and PKA, thereby activating Rim15p, which inactivates the stress response-associated transcription factors Msn2p and Msn4p [30]. This process includes the accumulation of glycogen. As these ICGCs are conditioned on *NSF1*, the abovementioned data in combination provide further evidence for the participation of Nsf1p in the response to fermentation stresses. 

#### *NSF1* and protein synthesis

The two most prevalent down-regulated functional categories of both the MFD and VFD ICGCs were the ribosome assembly/protein synthesis and transcription/translation regulation groups (Tables 1 and 2). This was supported by significant GO terms related to translation regulation and protein synthesis in both datasets (Tables S3 and S4). Strikingly, genes encoding the large and small ribosomal subunits, including *RPL7A*, *RPL2A*, *RPL18A*, *RPS17B*, *RPL8A, RPS3, RPS2, RPS9B* (Table 1) and, *RPL3*, *RPL34B*, *RPL22B*, *RPS5, RPS25A, RPS0A* (Table 2), and several GO terms related to protein synthesis (Table S3 and S4) were down-regulated in both datasets. Some of the GO terms included GO:0002181 (p-value < 1x10^-30^) and GO:0006414 (p-value 4.70x10^-06^).

The synthesis of ribosomal proteins and consequently the translational machinery is known to decrease toward the end of fermentation and also in response to nutrient limitation [7,31,32]. Due to the stressful environment the yeast did not multiply late in the fermentation as it does in the less stressful earlier stages. These results suggest that *NSF1* seems to be involved in down-regulation of protein synthesis as the fermentation proceeds. Whether *NSF1* is directly involved in the regulation of ribosomal gene expression or simply controlled by the same mechanism that controls ribosomal gene expression, is not known.

#### *NSF1* and sulfur metabolism

The sulfur metabolism-related genes *HOM2,* FOL *1* and *MET4* correlated with *NSF1* in the ICGCs of both datasets (Tables 1 and 2). *MET4* is the key regulator of the sulfur amino acid biosynthetic pathway, whereas *HOM2* is needed for the synthesis of L-aspartate-semialdehyde, the precursor of homoserine, which is needed for the production of the sulfur containing amino acids methionine and cysteine [33,34]. FOL *1* encodes a multifunctional enzyme essential in the biosynthesis of folic acid [35], which is readily converted to tetrahydrofolate, a methyl donor in the metabolism of glycine, methionine, serine and homocysteine [35]. *NSF1* involvement in sulfur metabolism was further suggested by the presence of several sulfur-related GO terms in the ICGCs of both the MFD and VFD datasets. Specifically, we identified GO terms associated with sulphur metabolic pathways and the biosynthesis and catabolism of the sulfur containing amino acids methionine and cysteine. Other sulfur metabolism-related GO terms were associated with sulfur compound biosynthesis (GO:004427 p=0.049) central to the well studied sulfur metabolic pathways in *S. cerevisiae* (Table S3). Collectively, these data suggest *NSF1* is involved in sulfur metabolism during fermentation.

### Biological data confirm *NSF1* as a negative regulator in sulfur metabolism

The observations made from the respective ICGCs led to predictions for the potential functions of *NSF1* during fermentation. To validate one of these predictions, we employed targeted molecular and cellular biology approaches to investigate the proposed function of *NSF1* in the sulfur metabolism of wine yeast.

#### *NSF1* is needed for the expression of sulfur metabolic genes near the end of Riesling fermentation

To gain insight into the impact of Nsf1p on the transcriptional response, we analyzed the differences in gene expression between the M2 and M2 *nsf1∆* strains near the end of the fermentation at 85% sugars fermented time point (Figure 3A) as this is when the transcription of *NSF1* is reportedly activated in the FSR gene group reported by [7]. A total of 934 differentially expressed genes (DEGs) at a 95% confidence level were identified; 497 were up-regulated and 437 were down-regulated in M2 *nsf1∆* with respect to M2. Strikingly, the DEGs contained ten sulfur metabolism genes all up-regulated in the mutant, including *MET5*, *MET8*, *MET14*, *MET16*, *MET17*, *MET22*, *MET28*, *MET32*, *MMP1* and *SUL2* (Figure 3B). *SUL2* and *MMP1* encode transporters of sulfur compounds [36,37], while *MET5*, *MET14*, *MET16*, and *MET17* encode metabolic enzymes needed for assimilation of sulfur into homocysteine, the precursor for methionine and cysteine synthesis [38]. *MET28* and *MET32* encode regulatory proteins that assemble into a multi-protein complex along with Cbf1p, Met31p and Met4p, which binds to conserved DNA elements (CDEIs) in the promoter regions of the *MET* genes to activate their transcription [39]. All these sulfur metabolism-related genes were up-regulated in M2 *nsf1∆* compared to the parent strain, suggesting that Nsf1p functions as a negative regulator of the *MET* genes (Figure 3B).

#### *NSF1* transcription is activated in sulfur limiting conditions in a Met4p-dependent manner

**Figure 6:**
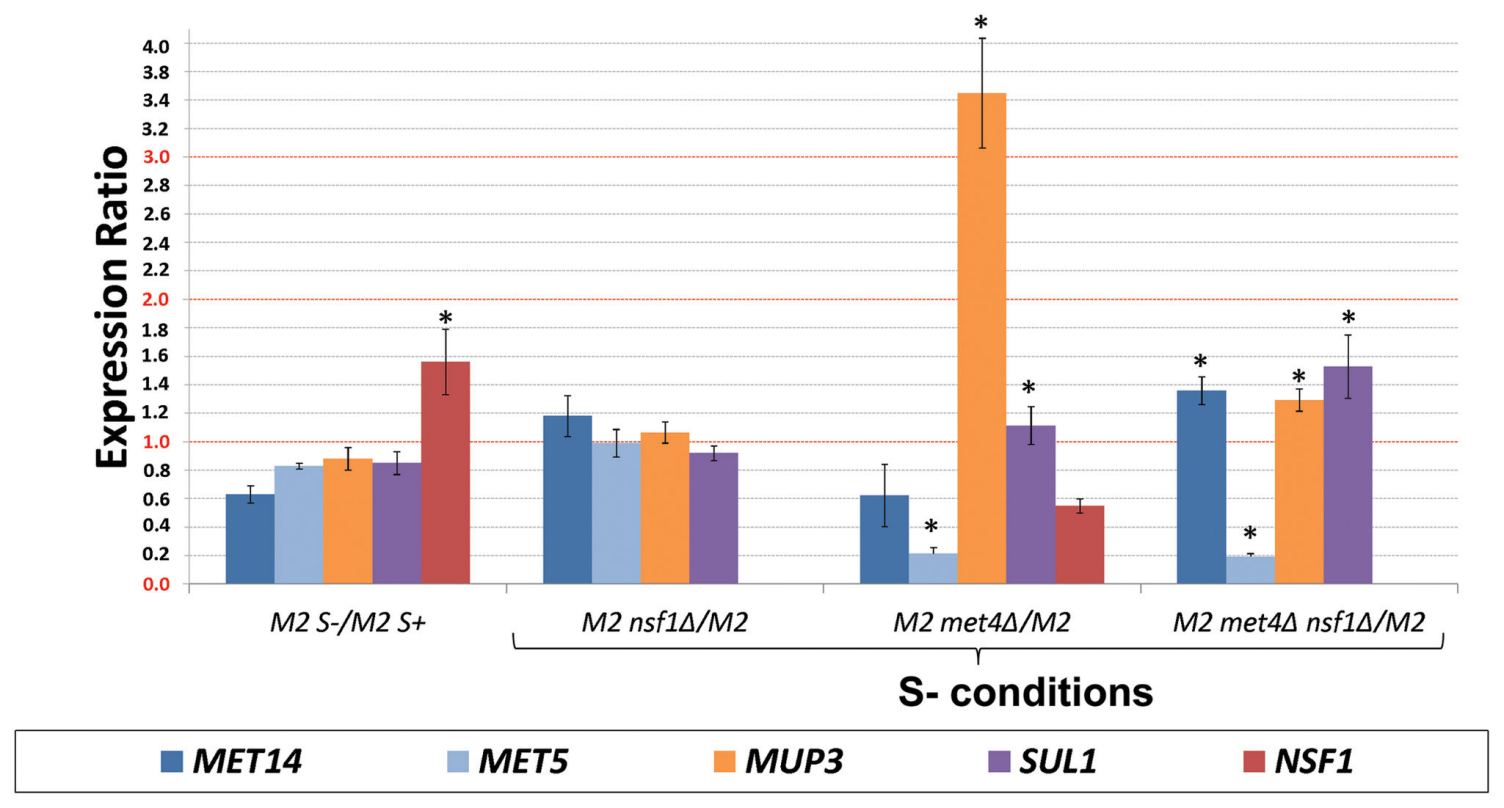
*NSF1* was needed for the controlled transcription of some sulfur pathway genes under defined sulfur conditions. The indicated genes were assayed in M2, M2 *nsf1∆*, *met4∆* and *nsf1∆met4∆* mutants under sulfur rich (S+) and (S-) limiting conditions. Asterisk (*) denotes statistically significant differences in gene expression at 95% significance level according to one sample t-test with population mean = 1 (no change in gene expression between assayed conditions).

Met4p is the major transcriptional activator of the sulfur metabolic genes [40]. We identified *MET4* as one of the ICGC genes in both the MFD and VFD datasets and showed that the sulfur metabolic genes are transcribed in a manner dependent on Nsf1p (Table 1; Figure 3B). To further investigate the relationship among Nsf1p, the regulation of sulfur metabolism and Met4p, we determined if *NSF1* transcription is affected by the reigning sulfur conditions in the medium. RNAs were extracted from cells grown in synthetic medium containing or devoid of MgSO_4_ as the sole sulfur source and analyzed for *NSF1* expression. The absence of sulfur increased the transcriptional activation of *NSF1* by 56% (Figure 6). When this transcriptional response was analyzed in the absence of *MET4* the expression level of *NSF1* decreased by 45% (Figure 6). *NSF1* transcription was therefore activated during sulfur limiting conditions in a manner dependent on Met4p. This finding confirms a functional link between *NSF1* and *MET4*, and provides the biological evidence for identification of new functions for poorly characterized genes using the ICC method.

We performed further gene expression studies in sulfur limiting conditions to identify possible co-regulatory roles for Nsf1p and Met4p in sulfur-regulated gene expression. The gene expression levels of *MET14*, *MET5*, and *SUL1* were monitored in the parent strain (M2), *nsf1∆*, *met4∆* and *nsf1∆met4∆* strains. Overall, the results indicated the greatest gene expression variation in a single *met4*Δ and double *nsf1*Δ*met4*Δ mutants compared to the parent M2 strain (Figure 6). Surprisingly, changes in expression of the genes analyzed were not statistically significant in the *nsf1*Δ single mutant grown in sulfur limiting conditions (Figure 6). By contrast, the transcription of *MET14* and *MET5* was clearly down-regulated, while that for *SUL1* was unchanged in the *met4*Δ mutant. However, the negative regulatory role of *NSF1* was clearly observed in the *nsf1*Δ*met4*Δ double mutant; in comparison to the *met4*Δ single mutant, the transcription of *MET14* and *SUL1* increased, while that of the *MET5* gene was unchanged. These observations suggest that the negative impact of *NSF1* on gene expression in sulfur limiting conditions could be masked by the presence of Met4p.

#### Sulfur conditions affect the sub-cellular localization of Nsf1p

**Figure 7:**
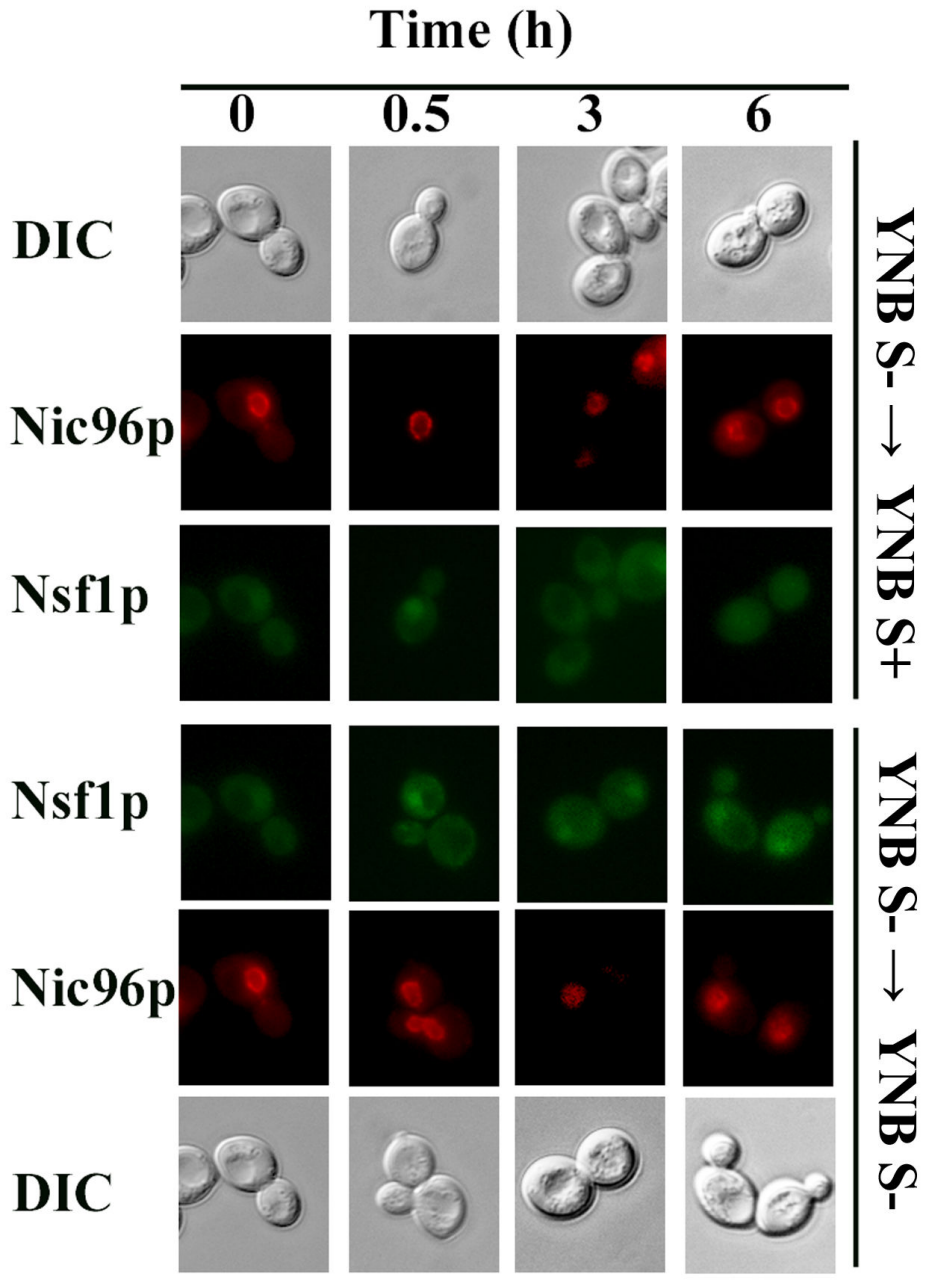
Nsf1 localized to the nucleus under limiting sulfur conditions. M2 *NSF1-GFP* cells transformed with pNIC96-mCherry-hphMX were pre-cultured in YNB S- medium to early log phase and shifted to fresh YNB S+ or YNB S- medium. Cells were monitored by fluorescence microscopy at the indicated times. The arrow (→) represents media shift.

**Figure 8:**
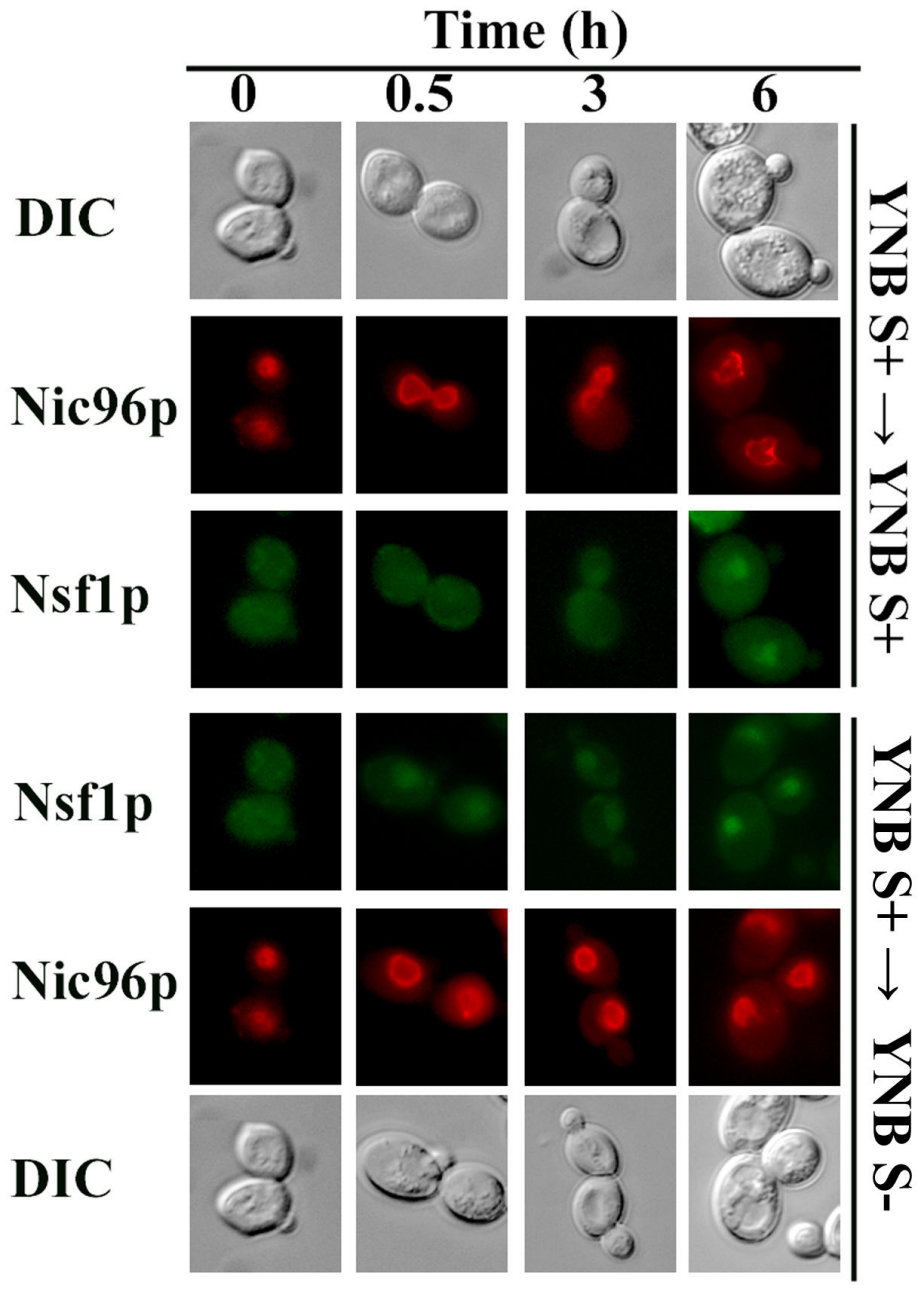
Nsf1 was not nuclear under rich sulfur conditions. M2 *NSF1-GFP* cells transformed with pNIC96-mCherry-hphMX were pre-cultured in YNB S- medium to early log phase and shifted to fresh YNB S+ or YNB S- medium. Cells were monitored by fluorescence microscopy at the indicated times. The arrow (→) represents medium shift.

Since Nsf1p is a transcription factor that is localized to the nucleus of the cell under salt stress and glucose limiting conditions [8], the sub-cellular localization of Nsf1p was investigated in sulfur rich and sulfur limiting conditions to further support its involvement in sulfur-regulated gene expression. Nsf1-GFP clearly co-localized to the nucleus with Nic96-mCherry in sulfur limiting conditions. When yeast cells were grown in and shifted to sulfur limiting media, Nsf1-GFP was nuclear throughout the entire time course (Figure 7). Interestingly, Nsf1-GFP was visible in the nucleus up to 30 min following a shift from sulfur limiting to sulfur rich conditions, but was absent from the nucleus 3 hours or longer after the shift (Figure 7). Also, when yeast cells were grown in sulfur rich media, Nsf1-GFP was not initially visible in the nucleus (Figure 7). When these cells were shifted to sulfur limiting media, Nsf1-GFP was visible in the nucleus 30 min after the shift. However, then cells were shifted to sulfur rich conditions, Nsf1-GFP was visible in the nucleus only at 6 h post-shift (Figure 8). It is important to note that glucose depletion stimulates Nsf1p entry into the nucleus [8]. Nsf1p nuclear localization 6 h after the shift from sulfur rich to sulfur rich conditions could therefore be due to the decreases in sulfur and/or glucose (Figure 8). This was not the case when cells were shifted from sulfur rich to sulfur poor conditions as Nsf1p already appeared in the nucleus 30 minutes after the shift while glucose was still abundant, indicating the nuclear localization in this case was due to sulfur limitation.

Together, these results suggest that the presence of Nsf1p in the nucleus is tightly controlled by sulfur availability in the environment; limited sulfur in the environment results in nuclear Nsf1p, while rich sulfur stimulates the loss of Nsf1p from the nucleus.

## Discussion

The overall objective of this study was to employ inter-disciplinary approaches of both the data mining and molecular biology fields to unravel the function of a poorly characterized gene. We developed and applied the ICC method to microarray data generated by two different industrial wine yeast strains during the fermentation of Riesling grape juice to gain insight into the function(s) of the poorly characterized gene *NSF1*. These computational analyses were followed by verification with targeted molecular and cellular biology experiments to underline the validity of the ICC method in predicting the function for *NSF1*. 

The ICC method represents complex data clearly as a weighted graph of genes focusing on intrinsic relationships existing among these genes, thereby providing a closer view of *in vivo* biological systems. Converting gene expression data into a graph allowed application of Graph Theory techniques such as the search of the largest maximally interconnected sub-graph (ICGC). Here the biggest strength of the ICC method came from the use of characteristically stringent criteria to generate the ICGC of co-expressed genes centered on *NSF1*; each additional gene inclusion into the growing ICGC needed to satisfy the connection threshold to all genes already present in the existing ICGC. These characteristics made the ICC method highly suitable for the analysis of very small and complex datasets with a limited number of replicas even if the expression profile of the target gene has high degree of similarity to other gene expression profiles.

The ICC method applies a combination of the graph theory and multivariate analysis on correlation values, taking into account corresponding dependence between variables (i.e. genes) that more closely mimics the biological reality of gene-gene interactions and regulatory mechanisms of gene transcription. Importantly, the ICC method does not depend on the multivariate normality distributions as the expression data is transformed in the network with posterior application of the graph methods with *a posteriori* intuitive interpretation of the results.

Compared to other gene expression exploratory multivariate methods such as Boolean networks [41], ordinary differential equations [42] and Bayesian-network approaches [43], ICC clearly stands out due to its reasonable scalability, ease of final results interpretability, suitability to situations of conditional gene expression in gene function elucidation studies and ability to capture relationships between continuous variables without loss of information. Amongst the mentioned methods the Dynamics Bayesian-network (DBNs) approaches are very promising, but suffer from important shortcomings including the requirement of relatively large datasets due to need of training dataset for the candidate network construction, and poor scalability of non-heuristic algorithm implementations [44]. Although reasonably good for regulatory network predictions, the DBNs fall short in the creation of large co-expression gene networks for the purposes of the gene function prediction.

In this study, we showed that the resulting ICGC allowed for the prediction, with biologically proven accuracy, of target gene function(s) and could be easily applied to investigate functions of other poorly characterized genes. To this end, the functional characterization of some genes found in each of the VFD and MFD ICGCs supported previously known functions of *NSF1*, including its involvement in the regulation of the carbon and thus energy metabolism [8]. More interestingly, new functions for *NSF1* that correlate well with the fermentation-related context of the generated microarray datasets were predicted. The genes present in the ICGCs provided *NSF1* with a co-expression functional neighbourhood, implicating *NSF1* in the general responses to nutrients, osmotic stress and toxins, regulation of carbon and energy metabolism in response to nutrient limitation/starvation, regulation of protein synthesis and transcription/translation control, vesicle trafficking and protein trafficking, and sulfur metabolism.

As the sulfur metabolism of yeast is of great interest to the wine industry, we employed transcriptional analysis of potential Nsf1p target genes and subcellular localization studies of Nsf1p to verify the ICC method’s prediction of the possible involvement of *NSF1* in the regulation of sulfur metabolism. Our gene expression analysis suggests that Nsf1p functions as a negative regulator of some sulfur metabolism related genes, specifically *MET14* and *SUL1*, under sulfur limitation conditions. *NSF1* expression was elevated and Nsf1p localized more readily to the nucleus under sulfur limiting conditions. Similarly, *NSF1* expression increased [7] and Nsf1 localized to the nucleus of wine yeast (data not shown) near the end of wine fermentations. These lines of evidence suggest that Nsf1p expression increases in sulfur limiting conditions and Nsf1p subsequently localizes to the nucleus to fine-tune the transcriptional activation of genes needed for the assimilation of available sulfur.

The transcriptional activation of many sulfur assimilatory genes is governed by Met4p [40]. Our expression analysis using the *nsf1∆met4∆* double mutant suggests that some, but not all, sulfur assimilatory genes were controlled by both Met4p and Nsf1p. Nsf1p could therefore function to fine tune the Met4p-mediated transcriptional activation in response to sulfur availability. We also showed that *NSF1* transcriptional activation was partially dependent on Met4p. In addition, analysis of the *NSF1* 5’ upstream non-coding region revealed a Cbf1p-Met4p-Met28p binding site (5’-TCACGGC-3’) 268 nt upstream of the *NSF1* ORF, thereby providing further evidence for the transcriptional regulation of the *NSF1* by Met4p. In turn, Met4p levels could also be controlled by Nsf1p since the promoter region of the *MET4* contains the CCCCT sequence, the STRE that corresponds to the Nsf1p DNA binding motif [9].

The proposed regulatory model between Met4p and Nsf1p is not novel as there are similar examples that exist in *S. cerevisiae*. For example, when yeast experiences poor nitrogen conditions, Gln3p acts as a major transcriptional activator of nitrogen-regulated genes needed for growth [33]. Dal80p, a repressor of Gln3p-mediated activation of nitrogen-regulated genes, is only expressed in poor nitrogen conditions. Thus, Gln3p and Dal80p act together to fine-tune the yeast’s responses to nitrogen availability [45]. Gln3p is needed for the transcriptional activation of *DAL80* when the yeast is grown under nitrogen limiting conditions. The transcriptional activator Gln3p is needed for the activation of the expression of its own repressor Dalp80p to fine tune the expression of Gln3p target genes. The same model seemed to apply to the relationship between Met4p and Nsf1p when it comes to the controlled expression of some *MET* genes under sulfur limitation. 

Our main contributions include the application of the ICC method to molecular biology data analysis needs. Although the focus of this paper was the *NSF1* gene, the ICC method could be used to investigate the function of any gene. Prior to this work, *NSF1* was poorly characterized and thought to be involved mainly in the regulation of gluconeogenesis and salt stress responses [8]. The ICC method has identified new potential functions for Nsf1p; we have confirmed that this protein is also needed for the regulation of sulfur assimilation.

## Supporting Information

Figure S1 **The pseuso-code used for mining for the largest ICGCs.**
(DOCX)

Table S1 **Genes of the largest ICGC mined from the MFD dataset.**
(XLSX)

Table S2 **Genes of the largest ICGC mined from the VFD dataset.**
(XLSX)

Table S3 **GO terms corresponding to the functional enrichment assessment of the ICGC of the MFD dataset.**
(XLSX)

Table S4 **GO terms corresponding to the functional enrichment assessment of the ICGC of the VFD dataset.**
(XLSX)

Table S5 **Yeast strains and their genotypes.**
(DOCX)

Table S6 ***MET4* disruption PCR primer sequences.**
(DOCX)

Table S7 **Growth Media composition.**
(DOCX)

Table S8 **qPCR primer sequences.**
(DOCX)
