# Supplementary material for: Functional Analyses of NSF1 in Wine Yeast Using Interconnected Correlation Clustering and Molecular Analyses
Source: PLoS One. 2013 Oct 9;8(10):e77192. doi: 10.1371/journal.pone.0077192 (PMC3793944; doi:10.1371/journal.pone.0077192)
Supplement: Figure S1 — The pseuso-code used for mining for the largest ICGCs. (DOCX) [file pone.0077192.s001.docx]

**Input:s**Normalized microarray data represented by *n* by *t* expression matrix *X*, where *n* is the number of genes and *t* is the number of time points.

**Output:s**the largest ICGC conditioned on the target gene

**1 FOR** *i* 🡪*1…n*

**2** *array_PCC*[i] = PCC (*X*[*gene_i_*], *X*[*target*]);

**3 FOR i**🡪*1…n*

**4 IF** *array_PCC*[*i*] > 0.95 **OR** *array_PCC*[*i*]<-0.95

**5** *select_array*[i] = *gene_i_*

**6 FOR** *i*🡪*1…n*

**7 FOR** j🡪*1..n*

**8 IF** PCC(*select_array*[*i*], *select_array*[*j*]) > 0.95

**9 OR** PCC(*select_array* [*i*], *select_array*[*j*]) < -0.95

**10** *array_A*[*i,j*] = 1

**11 ELSE**

**12** *array_A*[*i,j*] = 0

**13** MC=∅; X=∅; *N*= ∅

**14 Bron–Kerbosch (*select_array*,*X*, *N*,*array_A*)**

**15 RETURN** the largest maximal clique (ICGC)
