# Supplementary material for: Functional Analyses of NSF1 in Wine Yeast Using Interconnected Correlation Clustering and Molecular Analyses
Source: PLoS One. 2013 Oct 9;8(10):e77192. doi: 10.1371/journal.pone.0077192 (PMC3793944; doi:10.1371/journal.pone.0077192)
Supplement: Table S5 — Yeast strains and their genotypes. (DOCX) [file pone.0077192.s006.docx]

**Table S5:** Yeast strains and their genotypes.

| Strain name | Genotype |
| --- | --- |
| M2 | M2 *MAT*α/a |
| M2 *nsf1*∆ | M2 *MAT*a/α *nsf1*::*kanMX4*/*nsf1*::*kanMX4* |
| M2 *met4*∆ | M2 *MAT*a/α *met4*::*natMX6*/*met4*::*natMX6* |
| M2 *nsf1*∆*met4*∆ *nsf1*∆ | M2 *MAT*a/α *met4*::*natMX6*/*met4*::*natMX6* nsf1::kanMX4/nsf1::kanMX4 |
| M2 *NSF1-GFP* | M2 *MAT*a/α *NSF1*/*NSF1-GFP1*::*natMX6* |
