# Supplementary material for: Functional Analyses of NSF1 in Wine Yeast Using Interconnected Correlation Clustering and Molecular Analyses
Source: PLoS One. 2013 Oct 9;8(10):e77192. doi: 10.1371/journal.pone.0077192 (PMC3793944; doi:10.1371/journal.pone.0077192)
Supplement: Table S6 — MET4 disruption PCR primer sequences. (DOCX) [file pone.0077192.s007.docx]

**Table S6:** *MET4* disruption PCR primer sequences.

| **Primer ID** | **5’->3’ sequence** |
| --- | --- |
| **MET4_Forward disruption primer** | ATTGTGTGTCATCGGGCCACACAAGCATATTGCTTGAATTTTCTTTCATCGTTCAACTTAAATCCACCCAATcggatccccgggttaattaa |
| **MET4_Reverse disruption primer** | CGACGCGTTTTACTTATAGGCTAACAAAAAAATTTTAACGCGTTTGAATAGATGTGTGATGAATGAAGAATAgaattcgagctcg**a**tta**caa** |
| **MET4_Forward**  **Confirmation primer** | CTAACCTACTACTGTCTCAGAGCC |
| **MET4_Reverse**  **Confirmation primer** | TCCGATTCGTCGTCCGATT |

**Note:** CAPITAL letters represent homologous regions of the *MET4* while smaller case letters are regions that bind to the pFA6-natNT2 plasmid (Euroscarf accession #: P30346) harbouring NatMX resistance gene.
