# Supplementary material for: Functional Analyses of NSF1 in Wine Yeast Using Interconnected Correlation Clustering and Molecular Analyses
Source: PLoS One. 2013 Oct 9;8(10):e77192. doi: 10.1371/journal.pone.0077192 (PMC3793944; doi:10.1371/journal.pone.0077192)
Supplement: Table S7 — Growth Media composition. (DOCX) [file pone.0077192.s008.docx]

**Table S7:** Growth Media composition

| Media ID | Main Components |
| --- | --- |
| YPD | 1% (w/v) yeast extract, 2% (w/v) peptone, 2% (w/v) dextrose |
| YNB | Yeast Nitrogen Base powder without ammonium sulfate and amino acids (5 g/L), Dextrose (20 g/L), NH_4_Cl (5 g/L), dextrose (20 g/L), CSM (0.79 g/L) |
| S+ (sulfur rich) | NH_4_Cl (4 g/L), MgSO_4_·7H_2_O (0.05 g/L), KH_2_PO_4_(3.0 g/L), MgCl_2_(0.4 g/L) dextrose (42 g/L) |
| S- (sulfur poor) | NH_4_Cl (4 g/L), MgSO_4_·7H_2_O (0.005 g/L), KH_2_PO_4_(3.0 g/L), MgCl_2_(0.4 g/L) dextrose (42 g/L) |
| YNB S+ | Yeast Nitrogen Base (YNB) powder without ammonium sulfate and amino acids (5 g/L), MgSO_4_·7H_2_O (0.05 g/L), dextrose (20 g/L), NH_4_Cl (5 g/L) |
| YNB S- | Yeast Nitrogen Base powder without ammonium sulfate and amino acids (5 g/L), dextrose (20 g/L), NH_4_Cl (5 g/L) |
