# Supplementary material for: Functional Analyses of NSF1 in Wine Yeast Using Interconnected Correlation Clustering and Molecular Analyses
Source: PLoS One. 2013 Oct 9;8(10):e77192. doi: 10.1371/journal.pone.0077192 (PMC3793944; doi:10.1371/journal.pone.0077192)
Supplement: Table S8 — qPCR primer sequences. (DOCX) [file pone.0077192.s009.docx]

**Table S8:** qPCR primer sequences.

| **Gene name** | **Forward primer (5’🡪 3’)** | **Reverse primer (5’🡪 3’)** |
| --- | --- | --- |
| *NSF1* | CACCAATAGTGTAGCGGCATCA | TATCCGGTGCATTTGAACGTT |
| *MET14* | GGGAGGGTGTAATCAAGGAGTTT | CTGGTCGGTTCTCAAATGTAGCT |
| *MET5* | CATTGAGAGGAATGAATGCAGTTC | GGTAGAGCAGAAACCATGACATTTC |
| *MUP3* | CGGGATCTATCGTCTTCGGTAA | GACGTACTTGGACCACGAATCA |
| *SUL1* | TGAAAGTCGTCTGCCTGGATT | CAGATCAACCAGGCTTTGAACA |
